# Adverse reactions after treatment with dasatinib in chronic myeloid leukemia: Characteristics, potential mechanisms, and clinical management strategies

**DOI:** 10.3389/fonc.2023.1113462

**Published:** 2023-02-06

**Authors:** Fang Cheng, Qiling Xu, Qiang Li, Zheng Cui, Weiming Li, Fang Zeng

**Affiliations:** ^1^ Department of Pharmacy, Union Hospital, Tongji Medical College, Huazhong University of Science and Technology, Wuhan, China; ^2^ Hubei Province Clinical Research Center for Precision Medicine for Critical Illness, Wuhan, China; ^3^ Department of Hematology, Union Hospital, Tongji Medical College, Huazhong University of Science and Technology, Wuhan, China

**Keywords:** dasatinib, chronic myeloid leukemia, adverse reactions, pharmacotherapy, pleural effusion

## Abstract

Dasatinib, a second-generation tyrosine kinase inhibitor, is recommended as first-line treatment for patients newly diagnosed with chronic myeloid leukemia (CML) and second-line treatment for those who are resistant or intolerant to therapy with imatinib. Dasatinib is superior to imatinib in terms of clinical response; however, the potential pulmonary toxicities associated with dasatinib, such as pulmonary arterial hypertension and pleural effusion, may limit its clinical use. Appropriate management of dasatinib-related severe events is important for improving the quality of life and prognosis of patients with CML. This review summarizes current knowledge regarding the characteristics, potential mechanisms, and clinical management of adverse reactions occurring after treatment of CML with dasatinib.

## Case 1

A 62-year-old female with a pervious medical history of hypertension, type 2 diabetes mellitus, and dyslipidemia was diagnosed with chronic-phase CML. She received treatment with dasatinib 100 mg once daily. Which tests should be conducted regularly during follow-up?

## Case 2

A 71-year-old male, previously diagnosed with type II diabetes, hyperlipidemia, and coronary artery disease, was diagnosed with imatinib-resistant CML. He received treatment with dasatinib 50 mg once daily. Which cardiovascular tests should be performed prior to treatment? Which cardiovascular examination should be conducted during follow-up?

## 1 Introduction

Chronic myeloid leukemia (CML) is a type of cancer originating from the clonal proliferation of bone marrow hematopoietic stem cells. The disease is characterized by the oncogenic Philadelphia (Ph) chromosome carrying the *BCR-ABL* fusion gene (1). Imatinib was the first tyrosine kinase inhibitor (TKI) approved for the treatment of patients with Ph^+^ CML, which significantly improved the 5-year overall survival rate from 11% to 90% (2, 3). Second-generation TKIs are mainly designed to overcome resistance to imatinib; these agents have been associated with more rapid and profound molecular responses (4, 5). Dasatinib, a second-generation TKI, is increasingly proposed as first-line treatment for patients with CML (6, 7). Nevertheless, treatment with dasatinib has been linked to some uncommon adverse events (AEs), such as pleural effusion (PE) and pulmonary arterial hypertension (PAH) (8, 9). This narrative review summarizes current knowledge regarding the characteristics, potential mechanisms, and clinical management of AEs occurring after treatment of CML with dasatinib.

## 2 Methods

We searched for relevant full-text articles in the PubMed database using the following terms: “dasatinib”, “tyrosine kinase inhibitor”, “chronic myeloid leukemia”, “drug-related side effects*”, “side effects*”, “adverse reactions*”, “adverse drug reactions*”, “adverse drug events*”, “adverse events*”, and “drug toxicity”. These search terms were combined with the Boolean operators (AND/OR). All relevant case reports, clinical trials, and reviews were included. Articles published in languages other than English and those for which the full text was not available were excluded.

## 3 Clinical characteristic of dasatinib related AEs in patients with CML

Dasatinib-related AEs are mainly attributed to hematological and non-hematological toxicities. **Table 1** shows the characteristics of major clinical studies in this setting. Hematological toxicity is the most common AE associated with TKIs, and mainly manifested as leukopenia, neutropenia, anemia, and thrombocytopenia (**Table 2**). Serious hematological toxicity (grade 3/4) usually occurs within a few months after treatment; though rare, it is a major cause of dose reduction or treatment interruption. The administration of medication may be gradually resumed after remission. Non-hematological toxicity includes gastrointestinal toxicity, cutaneous reactions, musculoskeletal disorders, metabolic disorders, nephrotoxicity, pulmonary toxicity, and cardiovascular toxicity (**Table 2**). It mostly occurs at the time of treatment initiation and generally resolves without medical intervention. However, dasatinib-induced pulmonary toxicities (**Table 3**) require special attention, especially in the high-risk population.

**Table 1:** Characteristics of the major clinical studies of dasatinib treatment with CML patients.

Study	Treatment option	Treatment (N)				Follow time (years)
		Arm 1	Arm 2	Arm 3	Arm 4	
MDACC trail	First-line	Dasatinib (100mg daily, N=66)	Dasatinib (50mg bid, N=33)	NA	NA	1 y
DASISION trail	First-line	Dasatinib (100mg daily, N=258)	Imatinib (400 mg daily, N=258)	NA	NA	1 y
DASISION trail	First-line	Dasatinib (100mg daily, N=258)	Imatinib (400 mg daily, N=258)	NA	NA	2 y
DASISION trail	First-line	Dasatinib (100mg daily, N=258)	Imatinib (400mg daily, N=258)	NA	NA	5 y
NordCML006 trail	First-line	Dasatinib (100mg daily, N=22)	Imatinib (400mg daily, N=24)	NA	NA	3 y
START-R	Second-line	Dasatinib (70mg bid, N=101)	Imatinib (400mg bid, N=49)	NA	NA	2 y
CA180-034	Second-line	Dasatinib (100mg daily, N=167)	Dasatinib (50mg bid, N=168)	Dasatinib (140mg daily, N=167)	Dasatinib (70 mg bid, N=168)	2 y
CA180-034	Second-line	Dasatinib (100mg daily, N=167)	Dasatinib (50mg bid, N=168)	Dasatinib (140mg daily, N=167)	Dasatinib (70 mg bid, N=168)	7 y
DASCERN	Second-line	Dasatinib (100mg daily, N=174)	Imatinib (≥400mg daily, N=86)	NA	NA	2 y
Abhishek Maiti	First-line	Dasatinib (100mg daily, N=149)	NA	NA	NA	11 y

NA, Not applicable.

**Table 2:** Incidence of nonhematologic adverse events reported for dasatinib treatment.

Adverse events	Patients (%)			
	First-line		Second-line	
	Dasatinib 100mg daily		Dasatinib 100mg daily	
	Any Grade	Grade 3/4	Any Grade	Grade 3/4
Hematologic toxicity				
Neutropenia	7-64	6-29	12-64	12-63
Leukopenia	4	1	8-61	1-24
Anemia	13-90	3-13	23-91	6-20
Thrombocytopenia	18-70	9-22	23-65	11-57
Non-hematologic toxicity				
Gastrointestinal toxicity				
Diarrhea	17-59	0-5	9-37	0-3
Nausea	8-52	0-3	18-27	0-1
Vomiting	5-30	0-2	10 8 2 7	0-1
Abdominal pain	17	3	15 3 12	0-1
Musculoskeletal toxicity				
Muscle spasms	5-8	<1	1	0
Myalgia	6	<1	13-37.6	0-1.8
Musculoskeletal pain	11-69	<8	19-21	1-2
Cutaneous disorder				
Rash	11-27	0	8-32.8	0-3
Metabolic disorders				
Hyperlipidemia	NR	NR	NR	NR
Hyperglycemia	13	1	NR	NR
**Hepatotoxicity**	26	1	NR	NR
**Nephrotoxicity**	17	2	NR	NR
General Disorders				
Headache	12-48	0-5	15-33	1-2
Fatigue	8-76	1-13	2-37	0-4
Pulmonary disorders				
Pleural effusions	10-35	0-9	9-26	2-8
Pulmonary arterial hypertension	2.6-5	0-1	2.4	1.1
Cardiovascular Toxicity				
QTc prolongation	1.6-73	NR	NR	NR
Arterial vascular events	5	0-1	4	0-1

NR, not reported.

**Table 3:** Incidence of pulmonary disorders and cardiovascular toxicity reported in dasatinib treatment.

Pulmonary and cardiovascular toxicities	Patients (%)			
	First-line		Second-line	
	Dasatinib 100mg daily		Dasatinib 100mg daily	
	Any Grade	Grade 3/4	Any Grade	Grade 3/4
Pulmonary disorders				
Pleural effusions	10-35	0-9	9-26	2-8
Pulmonary arterial hypertension	2.6-5	<1	2.4	1.1
Cardiovascular Toxicity				
QTc prolongation	1.6-73	NR	NR	NR
Arterial vascular events	5	<1	4	<1

NR, not reported.

### 3.1 Hematologic toxicity, related mechanisms, and clinical management

In the MD Anderson Cancer Center (MDACC) trial, (10) dasatinib was used as first-line therapy for chronic-phase CML (CML-CP), and patients were randomized to receive 50 mg twice daily or 100 mg once daily. Most hematologic toxicities were temporary and mild; severe cases could be managed with emergency treatment disruptions and dosage reductions. The incidence rates of grade 3/4 neutropenia, anemia, and thrombocytopenia were 21% (n=13), 6% (n=4), and 10% (n=6), respectively. All grade 4 hematological toxicities (i.e., two cases of neutropenia and three cases of thrombocytopenia) occurred in the 50 mg twice daily group. The DASISION clinical trial (11) compared dasatinib (100 mg daily) with imatinib (400 mg daily). During the 12-month follow-up, most hematologic toxicities occurred within the first 4 months after treatment initiation. The incidence rates of grade 3/4 neutropenia, anemia, and thrombocytopenia were 21%, 10%, and 19% in the dasatinib group, and 7%, 10%, and 20% in the imatinib group, respectively. At 24 months, the rates of hematologic toxicity were similar to those recorded at 12 months (12). Moreover, at 5 years(13), there was no observation of new AEs. Grade 3/4 hematologic toxicity mostly occurred in the dasatinib group compared with the imatinib group (neutropenia: 29% vs. 24%; anemia: 13% vs. 9%; and thrombocytopenia: 22% vs. 14%, respectively). Maiti et al. performed a long-term follow-up study with dasatinib as initial therapy for patients with CML-CP (14). Hematological toxicities (any grade versus grade 3/4) were neutropenia (7% vs. 6%, respectively), leukopenia (4% vs. 1%, respectively), anemia (39% vs. 3%, respectively), and thrombocytopenia (27% vs. 11%, respectively). A network meta‐analysis was conducted to compare the safety profile of TKIs (i.e., ponatinib, bosutinib, dasatinib, nilotinib, imatinib, and radotinib) in patients with CML (15). The results showed that, among the examined TKIs, dasatinib was the least safe drug for CML and linked to a high risk of serious hematological toxicities. The surface under the cumulative ranking curve values for treatment with 140 mg dasatinib were 87.4%, 90.6%, 90.3%, and 97.2% for leucopenia, neutropenia, anemia, and thrombocytopenia, respectively.

There are two possible reasons associated with dasatinib-related hematologic toxicity. Firstly, Ph-negative cells were reserved by a weakened hematopoietic system compared with baseline, which is an inherent characteristic of high-risk patients. A previous study found that the development of hematologic toxicity during the first months of treatment with imatinib may be indicative of a worse long-term clinical outcome (16, 17). Secondly, dasatinib might play a role in several SRC kinase signal pathways, such as Lyn or Fyn, thereby inhibiting the downstream effect of platelet aggregation and megakaryocytopoiesis (18, 19). SRC kinases play an essential role in erythropoiesis and the survival of B cells and myeloid cells (20–22). In addition, dasatinib may induce eryptosis in human erythrocytes through Ca^2+^ channel loading and membrane permeabilization (23).

Most hematological toxicities are grade 1/2 and self-limiting, while grade 3/4 hematological toxicities are managed with dosage reduction or treatment discontinuation (5, 24). Therefore, at the initiation of treatment, blood testing is performed once per week during the first month, every 2 weeks in the second and third months, and every 3 months thereafter (25). In case of an absolute neutrophil count (ANC) <0.5 × 10^9^/L or platelet count <50 × 10^9^/L, the administration of dasatinib should be suspended. Treatment may be resumed when values for the ANC and platelet count reach ≥1 × 10^9^/L and ≥50 × 10^9^/L, respectively. Following the return of the ANC to the normal range within 2 weeks, dasatinib can be re-initiated at the original dose. In case of consistent decline in blood cells for >1 week after discontinuation of treatment with dasatinib, the dosage should be reduced to 70 mg or 50 mg once daily following a return within the normal range. In case of persistent neutropenia, the patient should be treated with growth factor. Although treatment with erythropoietin is effective against grade 3/4 anemia, recent guidelines do not recommend its use; instead, infusion of red blood cells is recommended (26, 27).

### 3.2 Gastrointestinal toxicity, related mechanisms, and management

Gastrointestinal AEs (e.g., diarrhea, nausea, vomiting, constipation, and abdominal pain) commonly occur after treatment with dasatinib. It has been reported that 30–50% of patients with CML experience gastrointestinal AEs (mostly grade 1/2) (28). In a recent clinical study of dasatinib as first-line therapy for patients with CML (14) (follow-up period: 11 years) the occurrence rate of nausea, vomiting, diarrhea, constipation, and abdominal pain was 52%, 30%, 59%, 34%, and 17%, respectively. In addition, the occurrence rate of grade 3/4 gastrointestinal AEs was <5%. Shah et al. carried out a 7-year follow-up study of treatment with dasatinib in patients with CML-CP who were resistant to imatinib or intolerant to 100 mg once daily compared with the other treatment arms (i.e., 140 mg once daily, 50 mg twice daily, and 70 mg twice daily) (CA180-034 trial) (29). The cumulative rates of gastrointestinal AEs were as follows: diarrhea (42% vs. 47%, respectively), and nausea/vomiting (27% vs. 43%, respectively); the AEs were mainly mild to moderate (grade 1/2). Furthermore, a recent meta-analysis evaluated differences in gastrointestinal AEs between different TKIs (30). The results showed that diarrhea (22.5%) was the most common symptom, followed by nausea (20.6%), and vomiting (12.9%). Also, there was significant difference in the average incidence of gastrointestinal AEs among TKIs: bostinib (52.9%), imatinib (24.2%), dasatinib (20.4%), and nilotinib (9.1%). Diarrhea was the most common gastrointestinal AE of dasatinib (28.1%). Nausea was the most common gastrointestinal AE of imatinib (33.0%) and nilotinib (13.2%). The incidence rate of grade 3/4 gastrointestinal AEs was ≤3%, except for severe diarrhea caused by bosutinib (9.5%).

A potential biological explanation of dasatinib-induced diarrhea is that the drug inhibits the stem cell factor receptor tyrosine kinase c-kit, which is highly expressed in gastrointestinal pacemaker cells (31, 32). Administration of dasatinib with food can improve symptoms of nausea and vomiting (33); treatment of nausea should be considered if the symptom persists. Supportive care, for instance, antidiarrheal agents (e.g., loperamide) with dietary modifications, should be given in case of severe or frequent diarrhea (34). Replacement with other TKIs or dose reduction, is recommended for patients with persistent severe gastrointestinal AEs and limited response to treatment (25).

### 3.3 Musculoskeletal toxicity, related mechanisms, and management

Dasatinib-induced musculoskeletal AEs mainly include muscle spasms, musculoskeletal pain, and myalgia. Previous studies showed that muscle spasms and myalgia were observed in 24% of patients treated with imatinib; however, these AEs occur less frequently with second-generation TKIs (35, 36). Musculoskeletal AEs were reported in 6% of patients receiving treatment with dasatinib, and the rate of grade 3/4 events was <1% (37). A long-term follow-up study showed that dasatinib-induced skeletal pain (e.g., joint pain, back pain, bone pain, limb pain, and chest wall pain) occurred in 103 patients (69%) (14). Muscle edema may be attributed to the inhibition of platelet-derived growth factor receptor beta (PDGFR-β) (38–40). However, whether PDGFR-β is associated with dasatinib-induced muscle cramps and myalgias remains unclear. Thus far, there are no drugs for the treatment of dasatinib-induced musculoskeletal AEs. Calcium supplementation can be considered to relieve muscle cramps, and bone and joint pain can be ameliorated by non-steroidal anti-inflammatory drugs (25).

### 3.4 Cutaneous reaction, related mechanisms, and management

Cutaneous toxicity is a common non-hematologic AE associated with TKIs. The incidence rate of cutaneous AEs in patients who received treatment with dasatinib was 11–27%(41–43). One phase I and five phase II clinical trials involving a total of 911 patients showed that the incidence of cutaneous toxicity of dasatinib was approximately 35%(44–46). In addition, the incidence of skin rash in patients with accelerated (22%) or chronic (13–27%) phase disease was higher than that recorded in patients with myeloid blast (11–14%) or lymphoid blast (15–17%) phase disease (47). Most events were grade 1/2 local and systemic erythema, macular and epidemic rash, or exfoliative rash. Of the patients, 16% had mucositis and/or stomatitis and 11% had pruritus. Dasatinib-induced rare cutaneous events were observed in individual case reports (46, 48): two patients with CML-CP who received dasatinib developed painful panniculitis. A case of possible small-vessel vasculitis by dasatinib-induced alveolitis has also been reported. However, a biopsy was not performed, and the AE resolved after intravenous administration of methyl prednisone (49).

The cutaneous toxicity of dasatinib is usually reversible, mild, and mostly self-limiting (50). For severe cases, the local use and short-term systemic administration of steroids can be considered to alleviate the symptoms. Severe or persistent manifestations may require dose reduction or temporary discontinuation of treatment. For skin-related symptoms, systemic corticosteroids should be used in conjunction with other supportive care. Following the resumption of treatment after temporary discontinuation due to serious skin events, steroids should be used at the beginning of re-introduction to prevent the recurrence of allergic reactions; the dosage can be gradually tapered after reaching the full dose. In some cases, a strategy involving the gradual increase of the dose may be required to achieve remission. Patients with fair skin should avoid direct exposure to sunlight or use sunscreen (41, 47, 50).

### 3.5 Metabolic disorders, mechanisms, and management

Generally, hyperglycemia and hyperlipidemia are rarely caused by dasatinib in patients with CML. Lu et al. compared the incidence of metabolic-related diseases in patients with CML who received imatinib, dasatinib, and nilotinib (51). They found that dasatinib was not significantly correlated with low/high blood glucose and hypertriglyceridemia. However, some studies have reported that dasatinib can lower the levels of blood glucose. Keiko et al. observed that dasatinib significantly improved hyperglycemia in a CML patient with diabetes (52). Franklin et al. evaluated the incidence rate of type 2 diabetes mellitus and hyperlipidemia in patients with CML who received dasatinib or nilotinib. The incidence rate of type 2 diabetes mellitus (dasatinib, n=1,272; nilotinib, n=732) and hyperlipidemia (dasatinib, n=845; nilotinib, n=435) was 1.76% and 4.64%, respectively (53). A previous study showed that dasatinib could increase the levels of peroxisome proliferative activated receptor gamma coactivator 1 alpha (PGC-1α) in adipose tissue, but exerted an adverse effect on glucose intolerance in obese mice (54). However, these effects have not been investigated in human studies.

The levels of lipids and glucose should be monitored periodically during the first year of dasatinib therapy, and assessed at least once per year during follow-up. Additionally, healthcare professionals should assist patients in developing a healthy lifestyle (i.e., avoidance of tobacco use, regular exercise, and adherence to a low-fat and low-carbohydrate diet). If intervention is warranted, patients with CML should be managed according to the relevant guidelines for the treatment of hyperlipidemia and diabetes (55).

### 3.6 Hepatotoxicity and nephrotoxicity, related mechanisms and management

#### 3.6.1 Hepatotoxicity

Previous studies have shown that elevation of alanine aminotransferase/aspartate aminotransferase levels may occur in 26% of patients treated with dasatinib and in <1% of severe cases(56, 57). Dasatinib-induced hyperbilirubinemia is rare in patients with CML-CP or accelerated phase disease (1%); nevertheless, it is more common in those with blast phase disease (4–5%) (58). A recent meta-analysis showed that bosutinib, nilotinib, or ponatinib were associated with a higher risk of hepatotoxicity than imatinib; however, dasatinib was not linked to a significantly increased risk (59). The mechanism of dasatinib-related hepatotoxicity remains unclear. Direct and indirect mitochondrial toxicity may play an important role in the mechanism underlying the hepatotoxicity induced by dasatinib and bonatinib (60). In addition, an *in vitro* study investigating drug-induced hepatotoxicity showed that treatment with TKIs upregulated bile acid synthesis and further altered bile acid uptake and excretion (61).

#### 3.6.2 Nephrotoxicity

Although preclinical and clinical trials failed to link dasatinib to the occurrence of severe nephrotoxicity, some rare cases of nephrotoxicity have been found in clinical practice. An analysis of the recently updated US Food and Drug Administration (FDA) Adverse Reporting System database indicated that dasatinib was related with a high risk of nephrotoxicity, especially protein glomerular disease, versus other TKIs (62). Although, three cases of acute renal failure have been reported, they were not definitively linked to dasatinib (63). A previous study showed that the incidence of proteinuria in patients treated with dasatinib was 18%, and grade 3/4 events were rare (64). To our knowledge, thus far, nine cases of nephrotic syndrome caused by dasatinib have been reported (three children, six adults) (65–72).

Biopsy specimens obtained from patients with dasatinib-related nephrotic proteinuria revealed pinocytosis; electron microscopy showed that these cases were characterized by reduced podocyte foot processes (67–70, 72). Podocytes are essential for the establishment of the glomerular blood urine filtration barrier, and damage to these cells leads to proteinuria (73). Nephrotic proteinuria associated with dasatinib may be attributed to the inhibition of vascular endothelial-derived growth factor (VEGF) production (74–76). VEGF is produced in podocytes and binds to VEGFR-2, thereby controlling the cytoskeleton and interstitial membrane of these cells(77). Therefore, the glomerular damage induced by dasatinib may be similar to that caused by other VEGF inhibitors. However, a recent study showed that dasatinib-associated nephrotoxicity was mainly related with its direct action on podocytes, rather than inhibition of VEGF (62). A previous study found that, following treatment with dasatinib, podocytes showed obvious molecular changes in adhesions, the actin cytoskeleton, and morphology (62, 78). Accumulating evidence confirms that dasatinib-induced glomerular toxicity directly affects the structural integrity of the podocyte cytoskeleton. This results in decreased cell elasticity and disruption of the key function of the podocyte cytoskeleton as a structural member of the filtration barrier (62, 79, 80).

Liver and renal function should be evaluated before initiating treatment with dasatinib. Moreover, routine testing should be performed during treatment. Dasatinib-induced hepatotoxicity and nephrotoxicity is usually reversible and self-limiting. Sustained significant or severe hepatotoxicity and nephrotoxicity may require more extensive evaluation. Such cases might be managed with dose reduction, temporary discontinuation of treatment, or replacement of dasatinib with other TKIs. In case of severe liver or renal injury, a specialist should be contacted for active treatment (81).

### 3.7 Pulmonary disorders, related mechanisms, and management

Pulmonary toxicities are unique AEs of dasatinib; the most commonly reported pulmonary toxicities are PE, PH, pulmonary edema, interstitial lung disease, pneumonia, chylothorax, etc. (82–85). Most of these AEs are clinically significant; hence, following the occurrence of these reactions, treatment discontinuation and additional medical intervention are required. Dasatinib-induced PE and PAH are discussed in more detail in the following sections.

#### 3.7.1 PE

PE has been observed after treatment with all *BCR-ABL1* TKIs, and dasatinib has been linked to the highest incidence (≤35%) (86–88). The frequency of occurrence, PE-related risk factors, and outcomes were assessed in two phase 3 clinical trials [i.e., DASISION (11) and 034/Dose optimization (29)] and in a pooled population (n=2,712) of 11 trials (12, 13, 29, 44, 45, 89–93) involving patients with CML and Ph^+^ acute lymphoblastic leukemia who received dasatinib therapy. PE was found in 6–9% and 5–15% of patients per year in the DASISION and 034/Dose optimization trials, respectively. During the minimum follow-up of 5 and 7 years, PE occurred in 28% and 33% of patients in the DASISION(13) and 034/Dose optimization trials (29), respectively. In a long-term follow-up study of 149 patients with CML who received dasatinib (14), the incidence of PE was 26%; 3% of cases experienced grade 3/4 events. Annually, PE occurred in 15% of patients in the first year of treatment, 2–5% in the following 3 years, and approximately 1–2% thereafter. Shah et al. reported that PE occurred in 6% of CML patients treated with dasatinib 50 mg daily (89). However, PE was not correlated with the overall response to dasatinib, progression-free survival, or overall survival in patients with CML (94). Furthermore, risk factors for PE were the initial dosage (140 mg vs. 100 mg), twice-daily regimen, hypercholesterolemia, hypertension, other pulmonary diseases, and a medical history of cardiovascular events or autoimmune diseases (7, 82, 95). Moreover, the accelerated and blast phases of disease were closely associated with the development of PE, especially in patients who received high-dose dasatinib (94). In addition, the most important risk factor for the development of PE in clinical studies is patient age, which may be involved with exposure to dasatinib (96–98). Notably, dasatinib-related PE was not found to be correlated with fluid retention (7).

The pathogenesis of dasatinib-related PE may be attributed to the inhibition of PDGFR expression in pericytes, which are involved in the regulation of angiogenesis (89). Moreover, it may be linked to the inhibition of Yes and SRC kinases, which are associated with pleural epithelial stability through the regulation of cell adhesion (99). Previous studies demonstrated that dasatinib was associated with lymphocytosis. Regarding the pharmacodynamic mechanism, dasatinib affects the ability to maintain pulmonary endothelial integrity in a dose-dependent manner by generating mitochondrial oxidative stress, inducing endothelial cell apoptosis, and impairing vascular permeability (100, 101). Recently, it has been proposed that the development of dasatinib-related PE is associated with changes in intercellular connectivity and the production of stress fibers and reactive oxygen species in the cytoplasm (102, 103).

Treatment interruption, dose reduction, and supportive care are recommended for the management of PE (94, 104, 105). Firstly, for the CML patients at high risk of developing PE, and respiratory failure and prior or concomitant pleuro-pulmonary disorders in primary care, one of the TKIs other than dasatinib should be chosen. In addition, initial treatment with half-dose dasatinib (50 mg/day) was suggested to be a safe option for patients with newly diagnosed CP-CML, which accompanied with more favorable clinical response and toxicity profile compared with the DASISION trial at a longer follow-up (106). Iurlo et al. (105) retrospectively collected 853 CML patients receiving dasatinib in both first-line and second-line of therapy. The results indicated that 70.4% of the PE events were observed in patients received 100 mg/day, and patients who have reached the MMR or DMR, dose reduction before PE development may help to reduce the incidence rate of PE. Furthermore, therapeutic drug monitoring (TDM)-guided dose adjustment may have potential benefits for dasatinib treatment in patients with CML. Rousselot et al. (97) found that TDM-guided dasatinib dose optimization was feasible and resulted in a significant reduction of PE events in the long-term treatment, without impairing MMR rate. Therefore, individual characteristics of the CML patients, comorbidities, toxicity profile, and physician-clinical center experience are among the critical factors to be comprehensively taken into account while deciding on the proper and personalized first-line dasatinib therapy (107).

**Figure 1:**
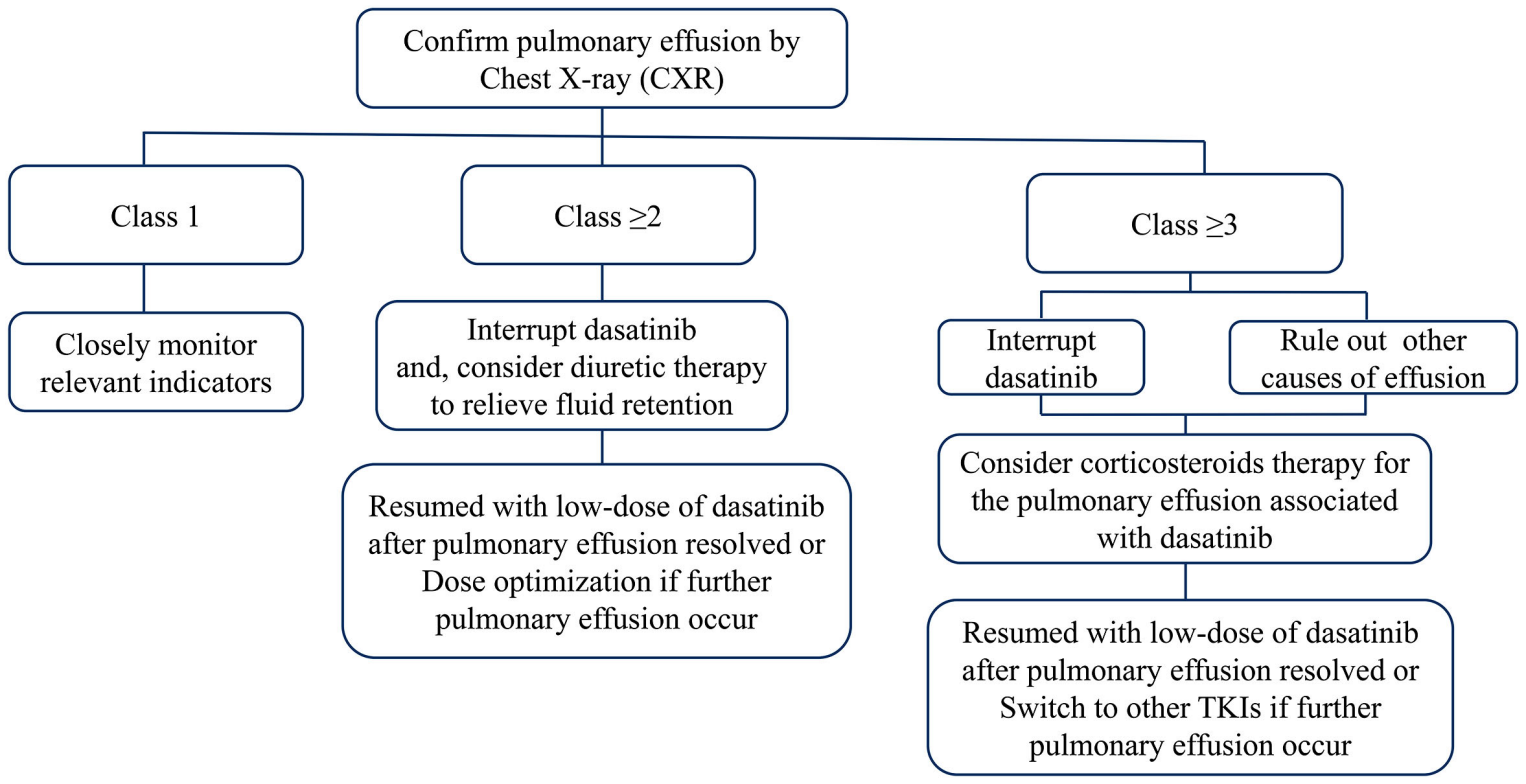
Clinical management of dasatinib-induced pulmonary effusion.

Early identification of PE by regular physical examination and chest radiography is crucial to avoid the development of more severe conditions. Intervention is not required for patients with grade 1 PE. For asymptomatic patients with grade ≥2 PE, the administration of dasatinib should be interrupted, and diuretic therapy could be initiated if fluid retention occurs. Dasatinib therapy can be resumed after the effusion has subsided. The dosage should also be reconsidered for the prevention of further episodes. For symptomatic patients with grade ≥2 PE, and asymptomatic patients with grade ≥3 PE, dasatinib therapy should be discontinued, and aggressive treatment with corticosteroids (40 mg daily for 4 days) should be initiated. Therapeutic thoracentesis should also be performed in severe cases. Dasatinib can be re-introduced following the resolution of the effusion. However, switching to other TKIs is recommended for the management of PE recurrence in severe cases (**Figure 1**).

Another approach for the management of PE depends on the severity and effusion volume. Cortes et al. (103) defined PE as: “small effusion” (effusion volume <500 mL with a blunting view of the costophrenic angle), “medium effusion” (opacity above the costophrenic angle), and “large effusion” (>30–50% effusion in half of the thorax). Asymptomatic patients with “small effusion” should be followed up regularly with chest X-ray (CXR) monitoring every 3 months in the first year and twice annually in the second year. For symptomatic patients, the frequency of CXR monitoring should be increased; dose reduction may be considered based on the treatment response, and CXR monitoring should be performed 1 month later. In case of persistent PE severity, CXR monitoring should be performed as described above. When the symptoms persist or worsen, the management is similar to that for medium/large effusions. Following the first episode of medium/large effusion, dasatinib should be immediately discontinued, and therapeutic thoracentesis should be performed. This should be followed by CXR monitoring every 2–4 weeks to assess the effusion volume until complete resolution. According to the clinical response of patients, dasatinib can be re-administered at a lower dose. Following the occurrence of more than two episodes, it is recommended to replace dasatinib with other TKIs.

#### 3.7.2 Pulmonary arterial hypertension

PAH is a life-threatening condition associated with long-term dasatinib therapy, especially in patients with PE (108, 109). In the absence of timely treatment, PAH may lead to right ventricular failure. Dasatinib-related reversible PAH was first reported in 2009(110). In 2012, nine cases of severe dasatinib-related PAH were described (111). According to the study conducted by Montani et.al, the estimated minimum annual incidence of dasatinib-related PAH in France is 0.45% (8). However, in a multicenter Australian study involving 212 patients who received dasatinib therapy, the estimated incidence of PH based on echocardiography was 5%; however, these cases were not confirmed by right-heart catheterization (112). As of December 31, 2017, the World Health Organization Pharmacovigilance Database (Vigibase^®^) included >440 cases of PAH associated with protein kinase inhibitors (8). In registry data, the overall incidence of dasatinib-related PH was <1%(113). In the DASISION trial (11), PH was suspected in 5% of patients based on echocardiography; nevertheless, only one patient underwent right-heart catheterization, which did not confirm the presence of PH. In the CA180-034 study (dasatinib as second-line therapy [n=670] in a 7-year follow-up) (29), PH of any grade was reported in 16 patients (2.4%); among those, grade 3/4 PH was detected in seven patients (1.1%). Of note, PAH was confirmed through right-heart catheterization in one patient. Furthermore, in a long-term follow-up study of dasatinib as initial therapy for patients with CML (n=149)(14), four patients with PE developed grade ≤2 PH, as detected by echocardiography and right-heart catheterization (two cases each).

The majority of patients who experienced PAH were female with history or present PE receiving long-term treatment with dasatinib(114, 115). Dasatinib-induced PAH mechanisms still remain unclear. It has been shown that dasatinib therapy increases the production of mitochondrial reactive oxygen species in a dose-dependent manner, further leading to endothelial apoptosis, pulmonary endothelial dysfunction, and PH (116). In addition, previous study indicated that chronic exposure to dasatinib may attenuate hypoxic pulmonary vasoconstriction responses and thus increase susceptibility to PAH, and seems to be independent of Src-inhibition (117–119). However, another reported mechanism underlying the effects of dasatinib is associated with SRC family kinases and PDGF pathways (120). SRC family kinases are responsible for cell proliferation in smooth muscle and reduce pulmonary arterial muscle tone. Inhibition of SRC family kinases leads to cell apoptosis and increased vascular resistance. Several studies have suggested that other signal pathways also contribute to endothelial dysfunction, leading to dasatinib-related PAH (99, 117, 121). Animal studies confirmed that dasatinib increased the biological activities of endothelial dysfunction markers (e.g., soluble vascular cell adhesion molecule 1 [VCAM-1], soluble intercellular adhesion molecule 1 [ICAM-1], and soluble E-selectin), leading to minimization of hypoxic vasoconstriction and impairment of endoplasmic reticulum function (117).

Given the relatively low incidence rate of dasatinib-related symptomatic PAH, systematic screening in asymptomatic patients is not readily feasible in the clinical setting. Transthoracic echocardiography is the preferred imaging technique for baseline risk stratification as it provides quantitative assessment of left and right ventricular function, chamber dilation, left ventricular hypertrophy, regional wall motion abnormalities, diastolic function, PAH, and pericardial disease, which may influence the therapeutic decision. Echocardiography could be performed as a reference baseline before initiating treatment with dasatinib to determine pre-existing PH. Following the occurrence of unexplained dyspnea with or without symptoms of right-heart dysfunction in the process of treatment, the first step is to evaluate the possibility of PH through echocardiography. Subsequently, examination through pulmonary function testing, CXR monitoring, and cardiac biomarker levels (e.g., troponin or N terminal pro-brain natriuretic peptide precursor) should be carried out to comprehensively assess the existence of other underlying conditions (e.g., PE, pneumonia, interstitial or obstructive pulmonary disease, ischemic heart disease, or left side heart failure). According to the current PAH guidelines, right-heart catheterization is recommended if echocardiography indicates an increased tricuspid regurgitation velocity >3.4 m/s, or <3.4 m/s with secondary PH signs (e.g., ventricular septum flattening or right ventricular dilation). High-resolution computed tomography or ventilatory perfusion scans should be performed to exclude other underlying causes of PH, such as pulmonary parenchymal disease or chronic thromboembolism. Notably, right-heart catheterization is essential for the accurate diagnosis and characterization of the hemodynamic profile. This is because dasatinib-induced PH may result in bilateral PE that could erroneously indicate post-capillary PH since it can be masked by left-heart disease. Patients treated with dasatinib may also have post-capillary PH due to elevated left cardiac pressure or increased PAH by overactivated circulation as a result of anemia or infection. Post-capillary PH is accompanied by high cardiac output volume and normal pulmonary vascular resistance(122, 123).

Treatment with dasatinib can be continued after confirmation of post-capillary PH or the hyperdynamic status through right-heart catheterization. However, following the confirmation of PAH, dasatinib should be immediately discontinued. For low-risk patients with mild symptoms, no right-heart failure, and low-risk hemodynamics, interruption of dasatinib therapy without intervention for PAH could be considered. Patients with moderate- or high-risk characteristics (e.g., severe symptoms, severe hemodynamic disturbances, or clinical symptoms of right-heart failure) should be proactively treated according to the guidelines for PAH (124, 125). Currently available medications for the treatment of PAH include phosphodiesterase type 5 inhibitor agents (e.g., sildenafil, tadalafil), soluble guanosine cyclase stimulants (e.g., riociguat), endothelin receptor antagonists (e.g., bosentan, macitentan), prostacyclin analogues (e.g., epoprostenol, traprost), and oral prostacyclin agonists (e.g., selexipag). Previous studies have reported that a combination of endothelin receptor antagonist and phosphodiesterase type 5 inhibitor can effectively treat TKI-induced PAH (108). Short-term re-assessment should be performed in all patients within 3–4 months of whether PAH related treatment is initiated. Given the high incidence of persistent PAH, invasive hemodynamic re-assessment should be performed wherever possible. Selection of the appropriate TKI depends on the severity of PAH and the stage of the underlying CML. Considering the likelihood of PAH deterioration or recurrence in this situation, patients should be closely monitored.

### 3.8 Cardiovascular toxicity, mechanisms, and management

#### 3.8.1 Corrected QT interval prolongation

Only one patient (0.4%) had QTc >500 ms in the DASISION trial; this finding may be attributed to the exclusion of patients with severe or even controlled cardiovascular disease (13). It has been reported that dasatinib moderately prolongs the QTc interval according to Fridericia (QTcF) by an average of +3 to +6 ms, and is less frequently associated with QTcF prolongation >500 ms (0.7%) or QTcF increase >60 ms (2.9%). However, there was no correlation between cumulative exposure and QTcF prolongation (126). An extensive meta-analysis of studies conducted up to 2018 indicated significant differences in the incidence of QTc prolongation induced by different TKI agents. QTc interval prolongation was more commonly observed with dasatinib (range: 1.6–73%) versus other TKIs (127). In addition, the US prescribing information for dasatinib advises caution in patients with increased risk of QT interval prolongation (128).

#### 3.8.2 Arterial vascular events

In October 2011, the US FDA issued a warning regarding the cardiopulmonary risks associated with dasatinib. It was recommended that patients using dasatinib should be monitored for signs and symptoms of cardiopulmonary disease before and during treatment with dasatinib (55). The 5-year follow-up of the DASISION trial(13) indicated an approximately 5% risk of an ischemic event in all patients, and two patients experienced transient ischemic attacks. A long-term follow-up study (14) showed that 13 patients (9%) had an arterial thrombotic event. These included nine cardiovascular events, comprising myocardial infarction (n=3), coronary artery disease (n=4), and chest pain (n=2); three cerebrovascular events (stroke or transient ischemic attack); and two cases of peripheral artery disease, including carotid stenosis (n=1) and peripheral artery disease together with coronary artery disease (n=1). The CA180-034 study (29) revealed an overall low incidence of ischemic events of all grades. The rate of cardiovascular ischemic events (e.g., myocardial infarction, angina, or coronary artery disease) was similar between the 100 mg daily group and all other dose groups (4% each). Main cardiac ischemic events reported in the 100 mg daily and the other dose groups were myocardial infarction (2% vs. 1%, respectively), angina (1% vs. 2%, respectively), and coronary artery disease (1% vs. <1%, respectively); the majority of ischemic events were grade 3/4. Furthermore, one patient experienced fatal myocardial infarction. Of the patients, 1% reported peripheral vascular events (all grades) in the other dosage groups (there were no events recorded in the 100 mg daily group), while <1% of patients reported grade 3/4 events. Cerebrovascular events (all grades) occurred in 3% and 1% of patients in the 100 mg daily and other dosage groups, respectively. Only 1% of the cerebrovascular events that occurred in the 100 mg daily group were grade 3/4. In contrast, all AEs recorded in other dosage groups were grade 3/4. A case/non-case study using AE reports registered in the US FDA Adverse Event Reporting System database compare the risk of cardiovascular events associated with TKIs. The results showed that dasatinib was associated with the highest incidence of heart failure and PH (129).

Clinical trials of dasatinib have yielded inconsistent results regarding the severity of cardiotoxicity. A statistical assessment showed that dasatinib did not significantly increase the risk of developing cardiovascular ischemia events in patients receiving the drug versus those with a similar condition (130). An analysis pooled the data obtained from the DASISION, READY, and 11 Phase I and II trials. The results demonstrated that the occurrence rate of ischemic events was 2–4%; however, most patients had a medical history or high-risk factors of atherosclerosis (128).

The mechanisms leading to cardiotoxicity are postulated to include mitochondrial dysfunction. Will et al. examined the effects of imatinib, dasatinib, sunitinib, and sorafenib on ATP content in H9c2 cells grown and respiratory capacity of isolated rat heart mitochondria. They found that only sorafenib directly impaired mitochondrial function at clinically relevant concentrations. For the other three kinase inhibitors lacking direct mitochondrial effects, altered kinase and other signaling pathways, are a more reasonable explanation for their cardiotoxicity (131). It is likely that toxicity is due to receptor kinase binding both on- and off-target. Five tyrosine kinase inhibitors (bosutinib, dasatinib, imatinib, nilotinib, and ponatinib) were evaluated in a neonatal rat myocyte model for their relative ability to induce myocyte damage (132). Results demonstrated that a lack of target selectivity was correlated with myocyte damage, but a correlation also existed with the strength of on-target Kd (dissociation constant).

Dexrazoxane is an anthracycline widely used as a cardiac protective agent; research has shown that this agent is ineffective in preventing dasatinib-induced damage to myocytes (133). Furthermore, treatment with dasatinib increased the accumulation of doxorubicin in myocytes, leading to damage. The mechanism underlying this effect may be associated with the ability of the drug to bind to one or more ATP-binding cassette-type efflux transporters(133, 134). Treatment with dasatinib also reduced the levels of phosphorylated extracellular signal-regulated kinase (ERK) in myocytes most likely by inhibiting RAF. It is well established that RAF/MEK/ERK is a pro-survival pathway. Thus, inhibition of this pathway by dasatinib suggests that this mechanism is involved in cardiovascular toxicity induced by this drug (133). Endothelial dysfunction was suggested to play an important role in dasatinib-induced cardiovascular toxicity (135–137). Moreover, Alsaad et al. found that dasatinib could induce hypertrophic markers in rat cardiomyocyte H9c2 cells through the aryl hydrocarbon receptor (AHR)-independent pathway (138), and Xu et al. revealed that dasatinib-induced cardiotoxicity acted *via* leading cardiomyocytes to High-mobility group box 1 (HMGB1)-mediated necroptosis(139), thereby suggesting other mechanisms may associated with dasatinib-induced cardiovascular toxicity.

Despite the low incidence of overall toxicity after treatment with dasatinib, such events can be life-threatening. According to 2022 ESC Guidelines on cardio-oncology (140), prior to the initiation of treatment with dasatinib, baseline cardiovascular risk assessment (including physical examination, blood pressure measurement, electrocardiogram, lipid profile, and HbA1c measurement) is recommended. In addition, baseline echocardiography is recommended in patients scheduled to receive dasatinib.

During the treatment, the use of drugs that may prolong the QT interval and strong cytochrome P450 family 3 subfamily A member 4 (CYP3A4) inhibitors that potentially increase drug accumulation and lead to life-threating cardiotoxicity should be avoided in patients treated with dasatinib. Extra caution should be exercised in patients receiving combination therapy with dasatinib and other cardiotoxic agents. Echocardiography may be considered every 6–12 months in patients who require long-term (>12 months) dasatinib therapy, and monitoring frequency should be considered every 3 months during the first year in high- and very high-risk patients. Importantly, clinicians should monitor the patients for any signs or symptoms of water and sodium retention, and intervene in time to reduce cardiac load and avoid cardiac insufficiency. Owing to the different effects of dasatinib on vascular diseases, the status of cardiovascular and metabolic conditions (e.g., hypertension, hyperlipidemia, and diabetes) should be evaluated multiple times after the initiation of treatment. The ankle-brachial index is a sensitive and specific method for the detection of systematic atherosclerosis. This index provides informative and noninvasive diagnosis for patients who initiate treatment with a specific TKI. When the ankle-brachial index value is beyond the normal range, duplex ultrasound must be conducted to identify the involved artery and atherosclerotic plaque; if necessary, angiography should be performed. Real-life data support the use of reduced doses of dasatinib to maintain optimal responses in patients initially treated with a full dose, with the aim of minimizing dose-dependent cardiovascular toxicity (119). However, following the occurrence of serious adverse cardiovascular reactions, dasatinib should be immediately discontinued. Cardiovascular events should be actively treated through collaboration with cardiologists, vascular medicine experts, or cardiac oncologists (141, 142).

## 4 Conclusion

Elucidation of the characteristics and potential mechanisms underlying the occurrence of dasatinib-associated AEs is crucial for the prevention, monitoring, and treatment of severe events. This would improve the clinical application of dasatinib and the quality of life of patients. The development of dasatinib-related AEs is linked to the therapeutic targets; hence, the distinction of therapeutic effects and toxicity is a major challenge for future research. Additional basic and clinical studies are warranted to gain deeper insight into the mechanisms underlying the dasatinib-induced toxicities. Such investigations may provide new directions for the discovery of novel *BCR/ABL1* TKIs.

## Author contributions

FC: Writing - Data Curation, Original Draft, Data Analysis. QX: Data Curation, Writing - Original Draft. QL: Data Curation, Writing- Original Draft. ZC: Data Curation. WL: Conceptualization, Visualization, Revised Draft.FZ: Project administration, Revised Draft. All authors contributed to the article and approved thesubmitted version.
